# Lithium metal batteries using a lithiophilic oxidative interfacial layer on the 3D porous metal alloy media†

**DOI:** 10.1039/d5ra00411j

**Published:** 2025-03-20

**Authors:** Yusong Choi, Tae-Young Ahn, Sang-Hyeon Ha, Hyungu Kang, Won Jun Ahn, Jae-In Lee, Eun-ji Yoo, Jae-Seong Yeo

**Affiliations:** a Defense Materials and Energy Development Center, Agency for Defense Development Yuseong P. O. Box 35 Daejeon 34060 Korea; b Department of Defense System Engineering, University of Science and Technology Daejeon 34113 Korea yusongchoi@ust.ac.kr +82-42-823-3400 +82-42-821-2457

## Abstract

Various lithium-infused metal anodes based on pure nickel foam, recognised for their superior properties, have been developed for application in lithium batteries. However, pure nickel foam exhibits significant reactivity with molten lithium during the infusion processes, such as coating and impregnation. In this study, a high-performance and ultra-stable lithium-infused metal anode (LI-NAFA) is synthesised through a simple oxidation treatment of nickel–chromium–aluminium (Ni–Cr–Al) alloy foam (NAF) at 900 °C in an air atmosphere. This approach effectively mitigates the material's reactivity with molten lithium, thereby enhancing the stability of the resulting anode. A layer of several hundred nanometers is generated, which converts the NAF surface from lithiophobic to lithiophilic. Additionally, the layers formed during oxidation enhance the molten lithium stability. A full cell test employing LI-NAFA showed stability during the molten lithium infusion and cycle performance. A full cell with pure lithium was also tested for comparison. The notable enhancement in performance can be ascribed to the excellent electrical conductivity of the NAF and improved cycling stability of lithium ions facilitated by uniform charge distribution. Following cell discharge, the LI-NAFA showed no formation of lithium dendrites and a reduction in dead lithium. LI-NAFA holds great potential for developing high-performance lithium metal batteries because of its favourable fabrication process and excellent cycling stability.

## Abstract 2

High-performance LI-NAFA was produced *via* oxidation of 3D nickel–aluminum–chrome (Ni alloy) based metal foam, which was then infused with molten Li.

## Graphical abstract

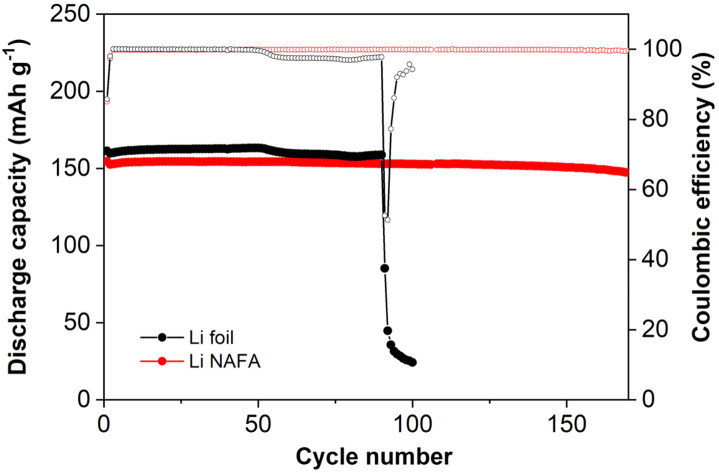

## Introduction

The growing demand for high-energy-density rechargeables fuels significant research into high-end lithium batteries.^1–9^ Lithium metal anodes have been extensively studied in the lithium battery field. Genuine Li can theoretically extract 3860 mA h g^−1^, showing a potential of −3.04 V against a standard hydrogen electrode.^10–18^ Li had many obstacles to overcome before it could be used as an anode for real battery applications; therefore, we tried to infuse the metal with molten Li to fabricate a Li-impregnated nickel alloy foam anode (LI-NAFA). Genuine Li exhibits bad cyclic performance because of dimensional instability during cycling and dendrite generation because of stripping and plating. Consequently, optimising the lithiophilicity against molten Li is a great breakthrough in the Li metal battery (LMB) research field. Theoretically, a substrate with high lithiophilicity is a prerequisite to achieving a good distribution of molten Li.^19–25^ Most materials in battery industries, such as metallic and carbonaceous substances, exhibit lithiophobic behaviour toward molten lithium. Poor wettability of molten lithium typically leads to liquid hemispheres forming on the substrate surfaces, making it unsuitable for direct infusion into metal anodes.^26–30^

Research has been devoted to porous metal anodes infused with molten lithium and anodes incorporating binder materials in recent years. Infusing molten lithium into highly porous pure nickel foam has emerged as a promising approach. Nevertheless, challenges associated with infusing molten lithium into porous structures with inherently lithiophobic surfaces present significant obstacles to its practical application in rechargeable lithium batteries.

Substantial wettability enhancements of molten lithium toward the porous nickel were documented.^21,22,26–28^ Generally, the accomplishment is by introducing a several tens or hundreds nanometre thick interlayer, which reduces the surface energy, such as Ag, Al, Al_2_O_3_, Si, organic functional coatings, CuO, ZnO, and Au. Various techniques, for instance, hydrothermal synthesis, vapour deposition, and atomic deposition, were applied to generate nanolayers on the surface of the substrate. However, these methods typically require high vacuum conditions, making them labour-intensive, time-consuming, and expensive. Consequently, it is imperative to fabricate more efficient and straightforward approaches to improve the wettability of molten lithium. Wu *et al.* introduced a thermal oxidation process to convert a lithiophobic copper surface into a lithiophilic one. However, this method requires 6 h to form a 635 nm thick Cu–O layer, and copper shows limited stability against molten lithium during infusion. Therefore, special precautions must be taken during molten lithium infusion, as nickel can easily melt in the presence of pure nickel foam when exposed to molten lithium.^29,30^

Pure nickel foam demonstrates significant reactivity with molten lithium during infusion processes, such as coating or impregnation. However, the least addressed issue regarding LMBs is the safety of lithium metal in elevated temperature environments caused by fire and short circuits (internal or external). Pure lithium in LMBs melts at 180 °C, implying that lithium can catastrophically melt down and catch fire, potentially causing an explosion. Therefore, countermeasures to prevent the meltdown of lithium in LMBs must be studied. Lithiophilic and stable 3D porous media, both mechanically and chemically, are prerequisites for making it real for the safe application of LMBs.

This study used nickel–chromium–aluminium (Ni–Cr–Al) alloy foam (NAF) as a substrate instead of nickel foam to produce a Li-impregnated nickel alloy foam anode (LI-NAFA). Cycle tests demonstrate the method's applicability in producing LI-NAFA. We consider this method one of the most efficient and practical approaches reported to date, with significant potential to facilitate the widespread implementation of porous metal anodes in rechargeable LMBs.

## Experimental

### LI-NAFA fabrication

NAF (with a porosity of 90%, pore size of 450 μm, and thickness of 1.6 mm; Alantum, Republic of Korea) was utilised in the study. Fig. S1† presents the control NAF, containing Ni, Al, and Cr, exhibited botryoid-like features. The NAF was fabricated with a cutter into 100 × 100 mm sheet form and then pressed to achieve a thickness between 0.2 and 0.9 mm (0.1 mm thickness gap) by adjusting the press (MH4389, Dong Jin Instrument, Republic of Korea). Ultrasonication of the pressed NAF samples was conducted in ethanol (Daejung, Republic of Korea, purity of 99.9% (anhydrous)) and in acetone (Daejung, Republic of Korea, purity of 99.8%) for 30 min for each sample. After the sample was left at 90 °C overnight, oxidation was conducted for the pressed NAF samples at 900 °C for 5 min in the air (Electronic furnace C-109, Hantech Co. Republic of Korea); this generates a lithiophilic oxidative lithiophilic layer on the NAF surface. The impregnation of lithium against the oxidised NAF samples was conducted in a glove box (Korea Kiyon, Republic of Korea, KK-021AD) with Ar-atmosphere strictly controlling the H_2_O or O_2_ one ppm content. In a glove box, Li (100 g, purity of over 99.9%) was melted in a 1000 mL Inconel vessel at 390 °C. The NAF samples were submerged in the molten Li and then removed to cool to room temperature. For comparison, as described before, the control NAF without the oxidative layer was impregnated with Li.

### Structural and chemical composition analysis

The microstructure and chemical compositions were investigated using transmission electron microscopy (TEM, Jeol (Japan), JEM-ARM200F) and scanning electron microscopy (FEI (USA), QUANTA-650). Chemical composition analysis was conducted with energy-dispersive spectroscopy (EDS, Bruker, Quantax 400).

### X-ray photoelectron spectroscopy (XPS) and X-ray diffraction (XRD) analyses

The surface chemistry of the oxidised NAF samples was examined using XPS (Thermo Fischer Scientific K-Alpha^+^, with a Kα 200 μm X-ray source). Before the XPS analysis, the surface was etched with argon. XRD analysis was conducted using D8-Advance (Bruker, Germany; radiation source Cu Kα [*λ* = 1.54 Å]).

### Molten lithium wettability

The control and oxidised foam samples were positioned on a plate within an Ar-filled glove box with H_2_O and O_2_ levels below one ppm. Molten lithium melted at 390 °C was then dropped onto the sample surfaces, and photographs were captured to measure the contact angle of the lithium droplets after deposition.

### Electrochemical measurements

The cycle performance was evaluated with a coin cell (CR2032) configuration. Before the molten lithium infusion, NAFA was roll-pressed to a thickness of 0.2 mm and trimmed to the disk-shaped anode (1.6 mm in diameter). For comparison, a genuine Li was fabricated as the same for the control anode. A cathode electrode consisting of LiNi_0.6_Co_0.2_Mn_0.2_O_2_ (NCM622, Umicore) was prepared by mixing the active material with carbon black (Super C, Timcal) and polyvinylidene fluoride (PVDF, Solvay) in *N*-methyl pyrrolidinone (NMP, 99.5%, Sigma-Aldrich) in a weight ratio of 90 : 5 : 5. The resulting blend was coated onto a 20 μm-thick aluminium foil and vacuum-dried at 100 °C for 12 h. The mass load and diameter of the cathode were 12.5 mg cm^−2^ and 1.2 mm, respectively. A 23.5 μm-thick ceramic-coated separator (Celgard, USA) material was applied as the separator to prevent internal short circuits of coin cells. The electrolyte consisted of 1.0 M lithium hexafluorophosphate (LiPF_6_), 2 wt% vinylene carbonate (VC), and 2 wt% fluoroethylene carbonate (FEC) in a solvent mixture of ethylene carbonate (EC) and ethyl methyl carbonate (EMC) with a 3 : 7 ratio by volume, purchased from Enchem Corp. (Republic of Korea). An electrolyte (100 μL) was dropped using a micropipette before sealing and left for at least 12 h for complete impregnation. Cycle tests were performed using a cycler (Maccor (USA), 4000 series) cycling between 4.2 and 3.0 V. During the cycle test, the samples were in an environment chamber controlling the temperature to 25 °C. The coin cell first operated in a CC-only formation cycle at a 0.1C rate (0.1 mA cm^−2^), followed by CC–CV cycles at a 0.2C rate (0.2 mA cm^−2^).

## Results and discussion

### Lithiophilicity of NAF

The weight variation of the foam was recorded during the lithium impregnation. After oxidation, the change in surface colour from silver to greenish-black is attributed to forming oxide materials following oxidation, as previously reported.^31,32^ The infusion of Li into the metal foam pores or impregnation must be conducted to fabricate an LI-NAFA. Fig. S2† shows that the pure nickel foam was highly unstable during the infusion or impregnation (see Fig. S2(b)†). To our knowledge, no reports discuss or suggest countermeasures for the instability of nickel foams during molten Li impregnation. A NAF, which is highly stable against Li metal and has good mechanical properties, was manufactured.^23^ However, this foam is highly lithiophobic. Therefore, we made the NAF lithiophilic using an efficient and simple method.

The developed oxide nanolayer addresses the issue of poor wettability and adjusts the surface energy of molten lithium, enabling the uniform spreading of metallic lithium on the NAF substrate.

**Fig. 1:**
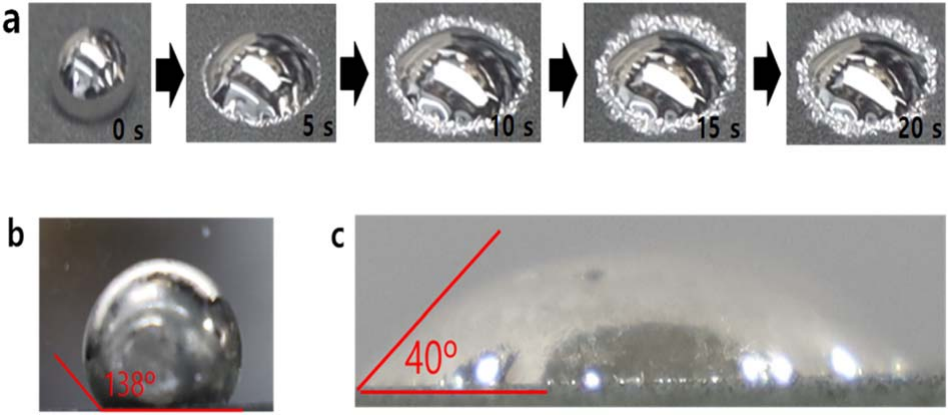
(a) Wetting behaviour of molten Li on the surface of the oxidised nickel–chromium–aluminium (Ni–Cr–Al) alloy foam (NAF) (pore size of 450 μm) relevant to time, (b) wetting angle without oxidation for NAF, (c) wetting angle of the oxidised at 900 °C sample.

The wetting behaviour on the surface of the oxidised NAF is shown in Fig. 1. The molten lithium was successfully infused to the oxidised NAF, a result attributed to the lithiophilic property of the oxides generated on the NAF surface. However, the control NAF without an oxide layer was also tested with a molten Li droplet, as shown in Fig. 1(b), which shows a lithiophobic property (wetting angle of 138°). A wetting angle greater than 90° implies that the surface is ultimately lithiophobic. Until the molten Li cooled down, it was not impregnated due to the poor wettability of the NAF; thus, no wetting angle change was observed at the beginning.

Contrastingly, the oxidised NAF (Fig. 1(c)) exhibits a significantly improved wetting angle of 40°, indicating excellent lithiophilic behaviour and good lithium impregnation. The wetting angle test results are presented in Table 1. Based on the wetting angle test, the oxidation method suggested in this study can drastically enhance the infusion of molten Li into the NAF. Previously reported high-performance nickel foam-based LI-NAFAs exhibited good wettability.^33^ The stability is inferior because of the reactivity between nickel and molten Li. However, the oxidised nickel alloy suggested in this study showed excellent stability and good wettability against molten Li.

**Table 1:** Summary of the wetting angle tests before and after oxidation for pure nickel foam and oxidised nickel–chromium–aluminium (Ni–Cr–Al) alloy foams (NAF)s

Foams	Performances			
	Stability during the impregnation	Li-impregnation	Time for complete impregnation vertically	
			After oxidation	Control
Pure nickel-foam	Poor	Good	7 s	Not wetted (meltdown in molten Li)
NAF	Stable	Good	10 s	Not wetted

**Fig. 2:**
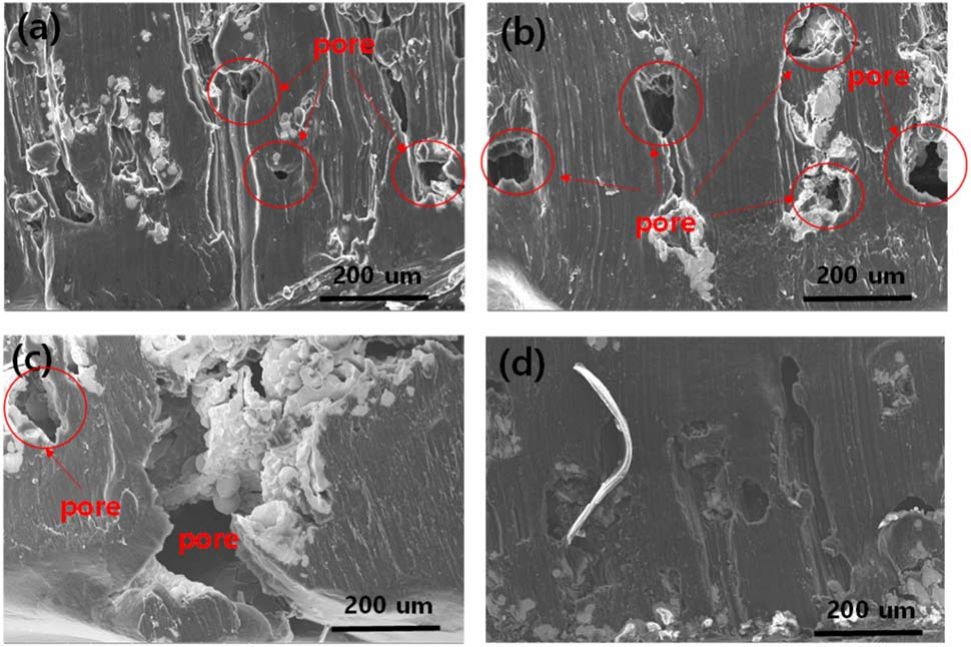
Cross-sectional scanning electron microscopy images of (a) control nickel–chromium–aluminium (Ni–Cr–Al) alloy foam (NAF) (pore size: 450 μm), (b) control NAF (pore size: 800 μm), (c) control NAF (pore size: 1200 μm), (d) oxidised NAF (pore size: 450 μm).

For a high-capacity anode, the full impregnation of molten lithium inside every pore of the metal foam is extremely important. Therefore, we investigated the intrusion mechanism of molten Li into the metal foam with and without oxidation. The impregnation behaviour of molten Li inside the oxidised NAF was also examined using cross-sectional scanning electron microscopy images of the oxidised and control NAFs after Li impregnation, as shown in Fig. 2. Fig. S1† presents the NAF image before lithium impregnation. Compared to images presented in Fig. 2, both control NAF and NAF after lithium impregnation exhibited structural modifications from their initial states (Fig. S1†). The impregnated Li was evenly distributed inside the oxidised NAF, as shown in Fig. 2(d), and thoroughly intruded into every pore. However, for the control NAF, as shown in Fig. 2(a)–(c), vacant pores were present for the control NAFs (with a pore size of 450, 800, and 1200 μm), which is ascribed to the poor wettability of the control NAFs. Hence, in addition to the wetting angle test, cross-sectional examination provides further evidence that applying oxide nanolayers on NAF is a useful and simple strategy for enhancing the lithiophilicity on the surface of NAF.

The Li content in the LI-NAFA, based on the thickness after pressing, was measured by weighing the mass variation during the Li impregnation, as shown in Fig. S3.† The lowest and highest Li contents were 13 wt% and 28 wt%, respectively, at 0.3 mm and 0.9 mm thickness. The maximum Li content of 28 wt% at a thickness of 0.9 mm in the oxidised NAF can deliver 386 mA h g^−1^, nearly equivalent to the graphite anode of 372 mA h g^−1^. The sound structure of the LI-NAFA can sustain its thickness during charging and discharging; therefore, LI-NAFA can represent excellent and stable cyclability as a next-generation high-performance LI-NAFA.

High electroconductivity is a prerequisite for applying LI-NAFA as a next-generation high-performance material. Therefore, the electroconductivity of the oxidised nickel alloy was investigated, as shown in Fig. S4.† The oxidation of nickel alloy generates an oxide layer by the reaction of oxygen in the air and the NAF at 900 °C. Most oxide layers are not electroconductive. Therefore, electroconductivity can be drastically reduced by the oxidation used in this study. The resistivity was investigated using the van der Pauw method, as shown in Fig. S4,† to examine the electroconductivity after the oxidation of the NAF. The van der Pauw method measures sheet resistivity by measuring the voltage difference when applying a current on one side of the sheet. The measured resistances before and after oxidation are listed in Table S1.† The resistance of the NAF after oxidation was 9.5 mΩ, whereas that of the control was 9.0 mΩ. No significant increase in resistance after oxidation was observed, attributed to the nickel-based oxide layer. Kim *et al.* and Kwon *et al.* reported that nickel oxide is conductive.^34,35^ Therefore, the nickel oxide layer formed during oxidation, and the Li-infused NAF exhibited excellent electrical conductivity. Thus, the oxidation method presented here is an effective approach for fabricating lithium metal anodes, making them highly suitable for real and safe high-performance lithium battery applications.

### Structure analysis of NAF

**Fig. 3:**
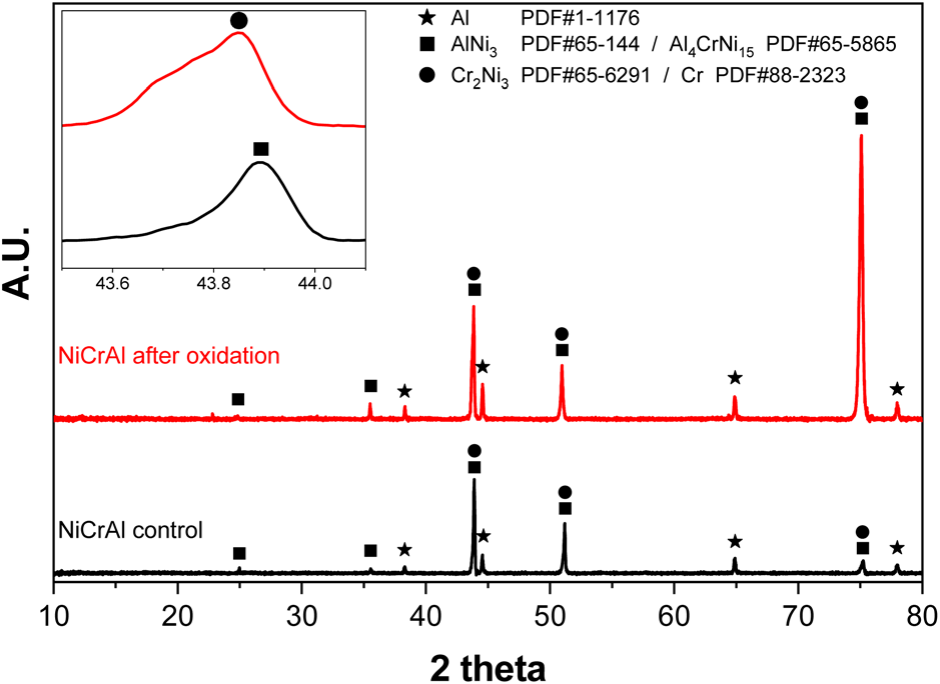
X-ray diffraction (XRD) pattern for the nickel–chromium–aluminium (Ni–Cr–Al) alloy foam (NAF) before and after oxidation.

As shown in Fig. 3, an XRD analysis was conducted to investigate the surface before and after oxidation. The XRD analysis both before and after oxidation showed peaks at 29.9°, 35.6°, 44.5°, 51.1°, and 75.3°, which were attributed to Cr (PDF-#1-1261), Ni (PDF-#1-1258), NiCrAl (PDF-#65-5865), AlNi_3_ (PDF-#65-144), Al_4_CrNi_15_ (PDF-#65-5865), and Cr_2_Ni_3_ (PDF-#65-6291). The inset Fig. 3 is a magnified view of the 43.8–44° region. Upon magnification, it can be confirmed that the AlNi_3_ and Cr_2_Ni_3_ phases coexist. The intensity of both 44.5° and 75.3° increases considerably, attributed to AlNi_3_ precipitation at the grain boundary during the oxidation at 900 °C.

No significant metal oxide crystal peaks are recognised after oxidation. The formation of the oxide layer on NAF after oxidation is more complex than that on pure nickel foam.^33^ In the earlier research regarding the pure nickel foam, the NiO peaks observed at 37.3°, 43.3°, 63°, 75.4°, and 79.4°, which are from the (111), (200), (220), (311), and (222) crystal planes, respectively, increased. The peaks became sharper, indicating enhanced crystallinity with increasing oxidation temperature^36^^.^

**Fig. 4:**
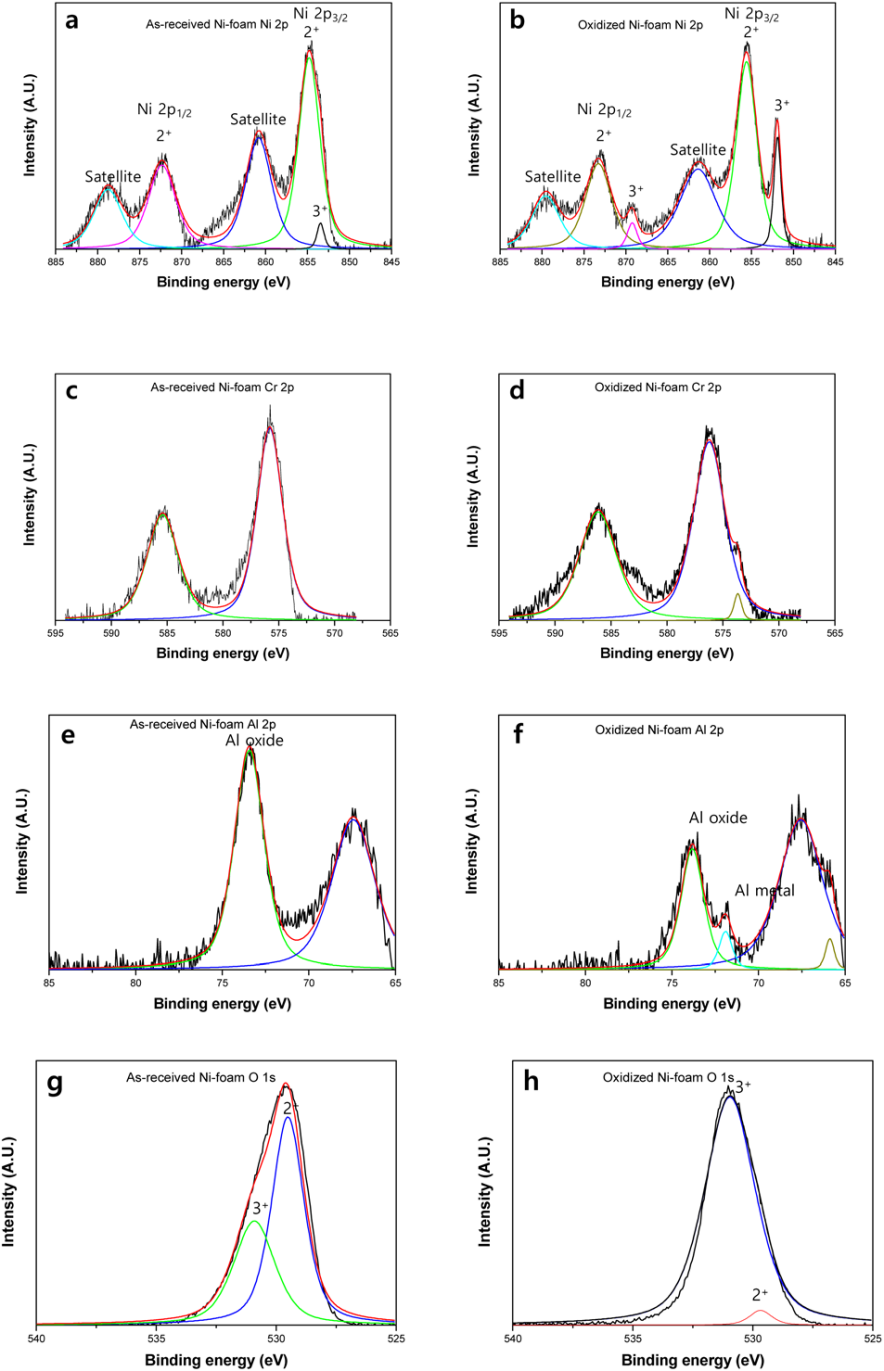
X-ray photoelectron spectra for the nickel–chromium–aluminium (Ni–Cr–Al) alloy foam (NAF) before and after oxidation. Ni 2p peaks of control (a) and oxidised (b), Cr 2p peaks of control (c) and oxidised (d), Al 2p peaks of control (e) and oxidised (f), O 1s peaks of control (g) and oxidised (h).

X-ray photoelectron spectroscopy (XPS) analysis was conducted to further study the chemical properties of the oxide layer formed on the oxidised NAF, as shown in Fig. 4. In contrast to the XRD analysis, the XPS analysis showed the coexistence of Ni^2+^ and Ni^3+^ species on the oxidised NAF at both control and oxidised NAF; this was attributed to the beam probing of only 0.37 nm of the surface layer, while the X-rays could analyse the bulk oxide layers. The surface Ni^2+^ content of NiO was shown in earlier XPS studies to result from oxygen chemisorption.^37^ As shown in Fig. 4(a), for the control NAF, the Ni–O^2+^ (529.6 eV) and Ni–O^3+^ peaks were attributed to the nickel oxide layer.^38,39^ The higher Ni^3+^ concentration on the surface of the oxidised NAF is a direct result of the thin oxidised layer before forming a crystalline Ni_2_O_3_ layer. Further investigation was conducted to elucidate the nickel oxide species generated on the surface of the NAF. The 855.6 and 861.2 eV peaks were attributed to Ni^3+^ and Ni^2+^, corresponding to Ni_2_O_3_ and NiO, respectively. The intensities of Ni^3+^ increased after oxidation compared to those of Ni^2+^.

Fig. 4(g) and (h) shows the O 1s peak of Ni_2_O_3_ at 531.2 eV. The O 1s peaks were analysed to elucidate the bonding properties between O and Ni; specifically, they relate to Ni^2+^ (O–Ni^2+^) at 529.6 eV and Ni^3+^ (O–Ni^3+^) at 531.4 eV. After oxidation, the intensity of the Ni–O^2+^ peaks, corresponding to that of NiO, increased significantly. This finding indicates that NiO was abundantly generated at this temperature, consistent with earlier research findings from XPS analysis of pure Ni foam oxidation.^33^ In the case of pure Ni foam oxidation, Ni_2_O_3_ and NiO were present after oxidation at a temperature lower than 900 °C. However, when the temperature was higher than 900 °C, NiO was preferentially generated on the surface of the Ni foam.^33^

**Fig. 5:**
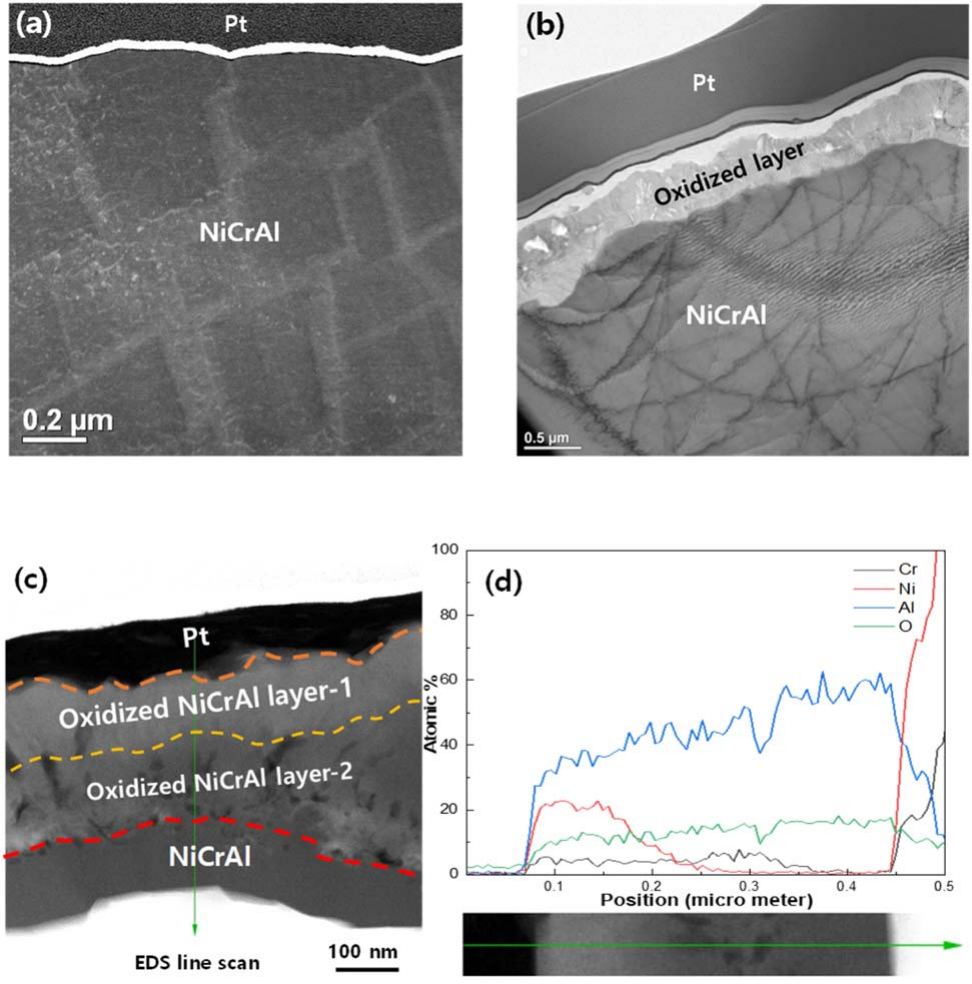
Transmission electron microscopy (TEM) analysis results for the nickel–chromium–aluminium (Ni–Cr–Al) alloy foam (NAF) before and after oxidation. (a) Scanning transmission electron microscopy (STEM) image of control NAF after FIB, (b) STEM image of oxidised NAF after FIB, (c) oxidised Ni alloy layers on the cross-sectional STEM image of oxidised NAF, (d) TEM-energy-dispersive spectroscopy (EDS) line scan result of oxidised NAF.

TEM analysis was conducted to investigate oxidation layers thoroughly, as shown in Fig. 5. Fig. 5(a) and (b) show the scanning transmission electron microscopy (STEM) images against control NAF after the focused ion beam (FIB) and the oxidised NAF after FIB. After oxidation, an Al and Ni alloy oxide layer of several hundred-nanometre thickness was formed. Based on Al 2p and O 1s XPS peaks and XRD results after oxidation of NAF, as shown in Fig. 3 and 4, the outmost surface of NAF consists of Al_2_O_3_, Ni_2_O_3_, Cr_2_O_3_, and precipitation of Al and Ni along the grain boundaries followed by the stable 2nd layer of Al_2_O_3_.

Fig. 5(a) and (b) show the STEM analysis results for the NAF before and after oxidation. The control NAF sample shows no oxidised layer between the matrix and Pt mounting layer; however, the oxidised layer, between 300 and 500 nm thick, is observable after oxidation. Fig. 5(c) and (d) show the oxidised Ni alloy layers in the STEM image and the TEM-EDS line-scan results of the oxidised NAF. The thickness of the oxide Ni and Al alloy layer-1 formed at oxidation temperatures of 900 °C, and the thickness of the oxide Ni alloy layer-2 was approximately 200 nm. Fig. 5(d) shows that the TEM-EDS line scan results identify the two alloy oxide layers and the matrix. The oxide layer on the Ni alloy matrix was clearly defined. We propose a mechanism for forming Ni alloy oxide layers based on the results of the analysis. Initially, the oxide layer of an approximately 200 nm thick Al and Ni alloy polycrystalline generated Ni_2_O_3_ and Cr_2_O_3_ layer (oxidised Ni alloy layer-1) was created between the oxidised Ni alloy layer-1 and Ni alloy scaffold followed by the Al_2_O_3_ layer (oxidised Ni alloy layer-2); as the oxidation proceeds from Ni–Cr–Al alloy → Al_*x*_Ni_*y*_, Al_*x*−*y*_Cr_*y*_Ni_*z*_, Ni_2_O_3_, Cr_2_O_3_ → Al_2_O_3_.

The main reason for using alloy foam is its mechanical properties. Among the mechanical properties, compressive strength is pivotal for application because the electrode is most likely to stack and pack to scale up to a high energy-density battery pack. As shown in Fig. S5(a) and (b),† the compressive strength and oxide layer weight gain of NAFA decrease and increase linearly, respectively, according to the logarithmic oxidation time. Less than 30 min of heat treatment is required for the optimum performance of NAFA from the perspective of both compressive strength and lithiophilicity. As the heat treatment duration increases, the compressive strength decreases with the increase in oxide layer thickness. Over 30 min of oxidation (a very thick oxide layer) can result in combustion during molten lithium impregnation because of a catastrophic exothermic chemical reaction between molten lithium and oxide (either NiO or Ni_2_O_3_).

The lithiophilicity against molten Li can be predicted by the Gibbs free energy (Δ*G*) calculated based on the density functional theory (DFT). Therefore, the Gibbs free energy was theoretically calculated using the DFT. Table S2† presents a comparison of the Gibbs free energy per unit area (Δ*G*_specific_) of the reaction between Li and a few promising coated materials, calculated following a previously reported method.^33^ The Δ*G*_specific_ of Li–Ni_2_O_3_ calculated using the VASP was −59.3 × *t* × 10^9^ J cm^−2^, indicating a negative value that is smaller than approximately three times that for ZnO (−20.0 × *t* × 10^9^ J cm^−2^) and slightly lower than the previously reported value for NiO–Li (−56.1 × *t* × 10^9^ J cm^−2^).^33^ Ni_2_O_3_–Li shows the best Δ*G*_specific_ value reported thus far. Based on the DFT calculation and XPS analysis, a significantly high Δ*G*_specific_ of the Li–Ni_2_O_3_ reactions generating Li_2_O results in a considerable improvement in molten Li infusion to oxidised Ni-foam.

### Electrochemical properties of LI-NAFA

**Fig. 6:**
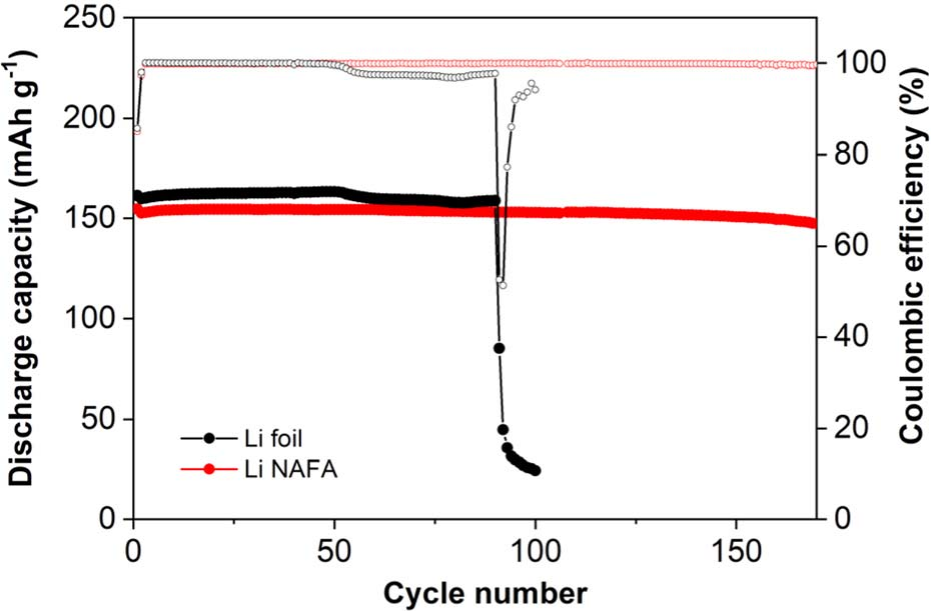
Full cell cycle performance of oxidised-nickel–chromium–aluminium (Ni–Cr–Al) alloy foam (NAF) and Li-foil full cell as a comparison (at 0.2C).

The full cell test was conducted using CR-2032 coin cell for Li-foil and Li-NAFA. The results are shown in Fig. 6. Pure Li foil and NMC full cell show a higher discharge capacity at the beginning of the cycle; however, passing the 50th cycle, discharge capacity decreases slightly, as does the coulombic efficiency. The slight decrease in the discharge capacity and coulombic efficiency are ascribed to the dead Li and soft short from the dendrite occurrence at the surface of the Li-foil. Eventually, at the 90th cycle, Li-foil full cell discharge capacity drops significantly. Unlike the Li-foil full cell, oxidised NAF full cells discharge stably over 170th cycles, as does the coulombic efficiency of 99.69%.

As shown in Fig. 6, using similar amounts of lithium, the Li-NAFA full cell exhibits higher cycle stability than the Li-foil full cell.

After the cycle test, the full cells were dismantled for post-mortem analysis. The dismantled anodes for Li foil and oxidised the NAF anode is shown in Fig. S6.† The surface of the Li foil anode after cycling was covered with black dead Li; however, the oxidised NAF anode after the cycle showed bright Li with grey staining in some parts of the surface, which was mossy like Li. The black dead Li was also observed at the control NAF anode after the cycle test, as shown in Fig. S7.† In the optical microscope image shown in Fig. S7(a) and (b),† the dead Li and the dendrite are distinctly shown.

Contrary to the Li foil and control NAF anode, the oxidised NAF anode shows no dendrite, and the dead Li shows no dendrite after the cycle over 200. The enhanced mechanical and chemical stabilities of the NAF against the molten Li and the lithiophilicity by oxidation were examined in this study. Based on the cycle test and post-mortem analysis, oxidised LI-NAFA mitigates the occurrence of dead Li and dendrite. The oxide layers formed on the surface of the NAF exhibited electrical conductivity, enabling electron transfer between the impregnated lithium and the NAF. The enhancing effect of electron conductivity within the NAF and the metal oxide layers significantly enhanced the stability of the system, as evidenced by the improved cycling performance of the LI-NAFA NAF; this resulted in a prominent enhancement in electrochemical properties when compared to that of the pure lithium anode. The NAF, developed *via* a practical and efficient oxidation process, demonstrated excellent electrochemical performance, indicating its suitability for lithium-ion batteries. Besides, the oxidation method is considered less time-consuming and economical, making it a viable approach for the massive scale (such as roll-to-roll) production of NAF anodes.

## Conclusions

A substantial enhancement in the wettability of molten lithium on the NAF was observed after subjecting the foam to an oxidation process in the air. This process formed a conductive oxide layer of thickness ranging between 300 and 500 nm. The oxide layer demonstrated high lithiophilicity, reducing the wetting angle of molten lithium on the NAF from 138° (in the control sample) to 40° (after oxidation). Additionally, cyclic testing of the LI-NAFAs revealed neither short circuits nor dendrite formation throughout the cycling process. It presents a valuable approach for advancing the development of rechargeable lithium batteries. The high-performance LI-NAFA was fabricated by oxidising an NAF, followed by the infusion of molten lithium. The oxidation process generated a highly lithiophilic and stable nanoscale oxide layer on the NAF surface, significantly improving molten lithium wettability and providing a simple yet efficient route for fabricating LI-NAFAs.

## Data availability

The authors confirm that the data supporting the article haven been included as part of the ESI.†

## Author contributions

Made substantial contributions to the conception and design of the study and performed data analysis and interpretation: Yusong Choi. Performed data acquisition: Tae-Young Ahn, Hyungu Kang, Eun-ji Yoo, Jae-Seong Yeo. Provided technical and material support: Sang Hyeon Ha, Won Jun Ahn, Jae In Lee. Wrote the first version of the manuscript: Yusong Choi, Tae-Young Ahn. All authors revised the manuscript.

## Conflicts of interest

All authors declared that there are no conflicts of interest.

## Supplementary Material

RA-015-D5RA00411J-s001
