# Supplementary material for: Lithium metal batteries using a lithiophilic oxidative interfacial layer on the 3D porous metal alloy media
Source: RSC Adv. 2025 Mar 20;15(11):8622–9. doi: 10.1039/d5ra00411j (PMC11923736; doi:10.1039/d5ra00411j)
Supplement: RA-015-D5RA00411J-s001 [file RA-015-D5RA00411J-s001.pdf]

Supplementary Information (SI) for RSC Advances.
This journal is © The Royal Society of Chemistry 2025

Supplementary Information

Lithium metal batteries using lithiophilic oxidative interfacial layer on the 3D porous metal alloy media

Yusong Choi[1,2]*, Tae-Young Ahn[1], Sang Hyeon Ha[1], Hyungu Kang[1], Won Jun Ahn[1], Jae In Lee[1], Eun-ji Yoo[1], Jae-Seong Yeo[1]

[1]Defense Materials and Energy Development Center, Agency for Defense Development, Yuseong P.O. Box 35, Daejeon, 34060, Korea

[2]Department of Defense System Engineering, University of Science and Technology, Daejeon 34113, Korea

*Corresponding author. yusongchoi@ust.ac.kr, Tel: +82-42-821-2457; Fax: +82-42-823-3400

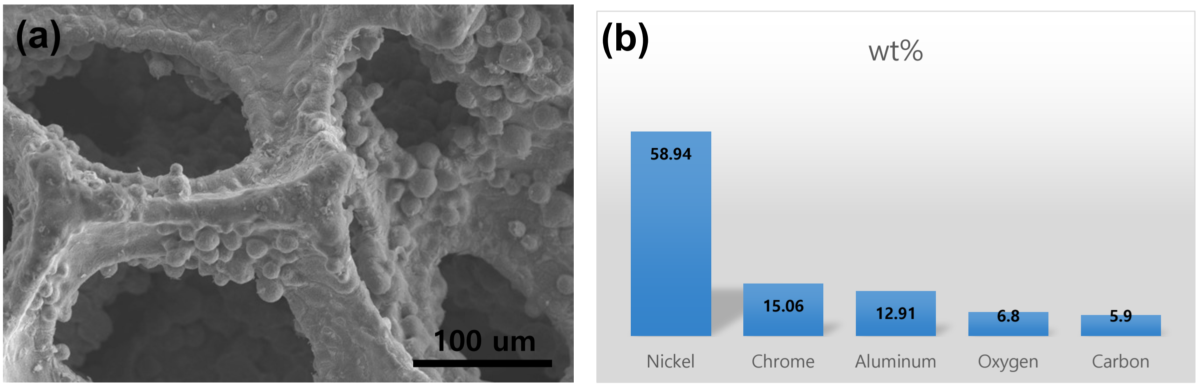

Fig. S1. (a) Scanning electron microscopy (SEM) images and (b) alloy composition ratio of the as-received NiCrAl foam

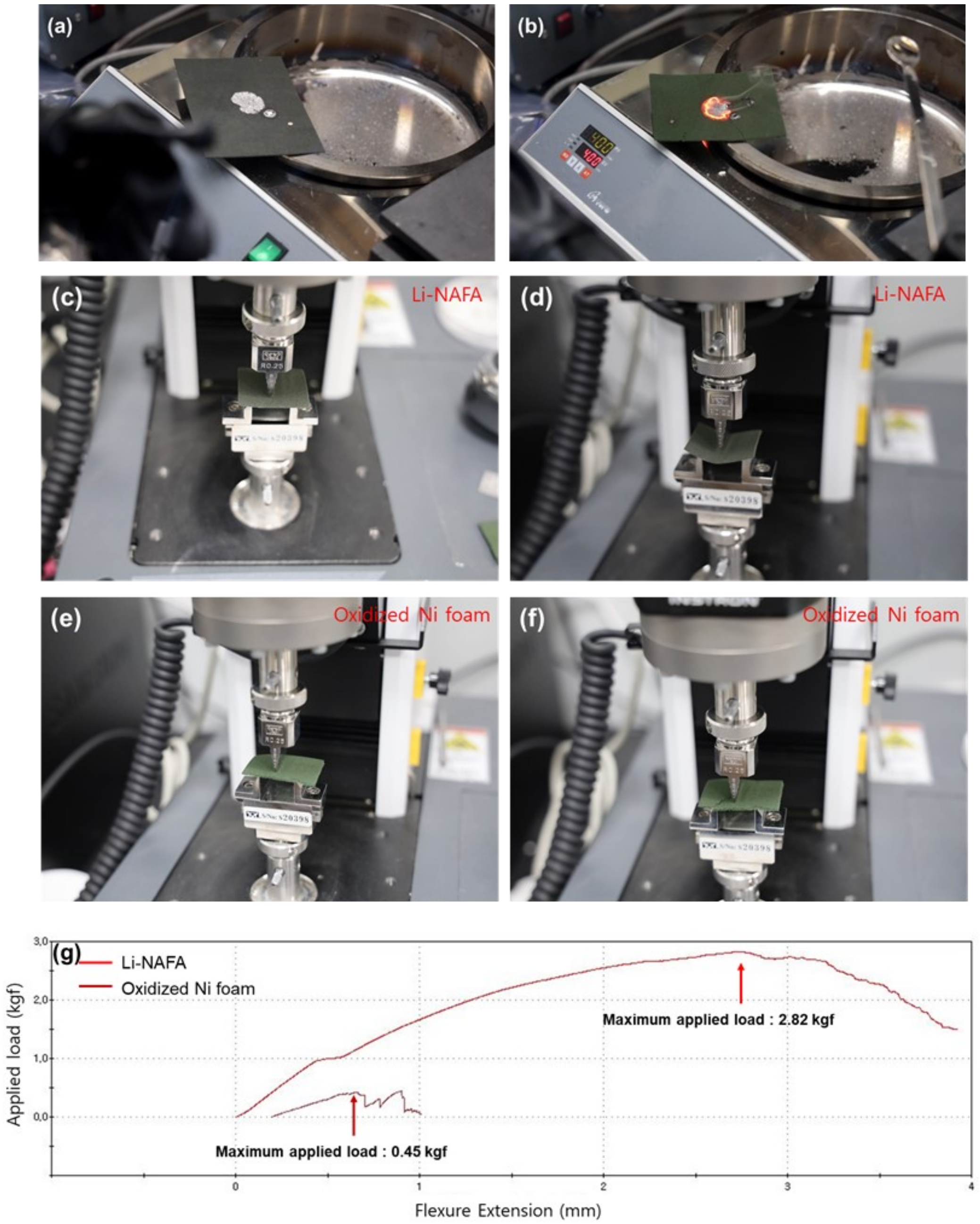

Fig. S2. Comparison of the infusion characteristics of lithium melted at 400 °C: (a) Li-NAFA (good molten lithium impregnation) and (b) oxidized Ni foam (Catch fire, burn out, and leave a hole). Bending test result comparison: images of Li-NAFA (c) pre-test and (d) post-test, and oxidized Ni foam (e) pre-test and (f) post-test (g). The bending test results show that the maximum applied load for Li-NAFA is 2.82 kgf, whereas that for oxidized Ni foam is 0.45 kgf**.**

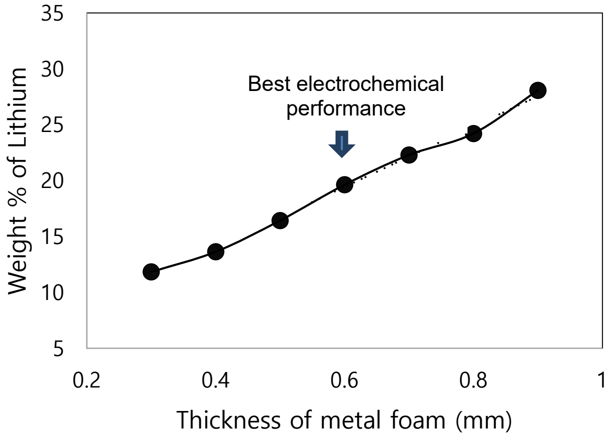

Fig. S3. Comparison of weight of lithium content according to the variation of the thickness of oxidised NAF. (An increase in the density of the foam directly corresponds to a reduction in the internal pore volume. Therefore, as the thickness increases, the overall lithium volume within the foam increases, leading to a reduction in the foam's overall volume fraction. As confirmed in our study, the optimal foam thickness for achieving the best electrochemical performance is 0.6 t.)

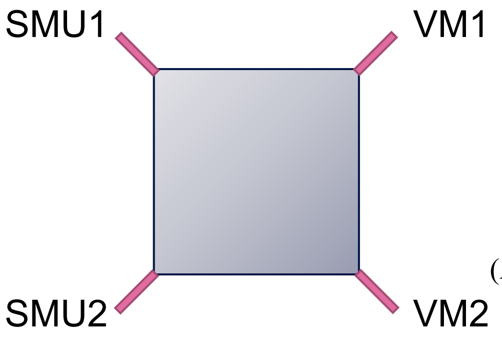

Sheet resistance

$$\rho_s = \frac{\pi}{ln2} \circ \frac{V}{I} = 4.53 * \frac{V}{I}$$

Where, I (A): current difference between SMU1 and SMU2

V. (V): voltage difference between VM1 and VM2 ρs (Ω): sheet resistance

Fig. S4. Resistivity measurement by according to Van Der Pauw.

Table S1. Resistivity measurement result for control NAF and after oxidation of NAF according to Van Der Pauw

Resistance control (mΩ)	Resistance after oxidation (mΩ)
9.0	9.5

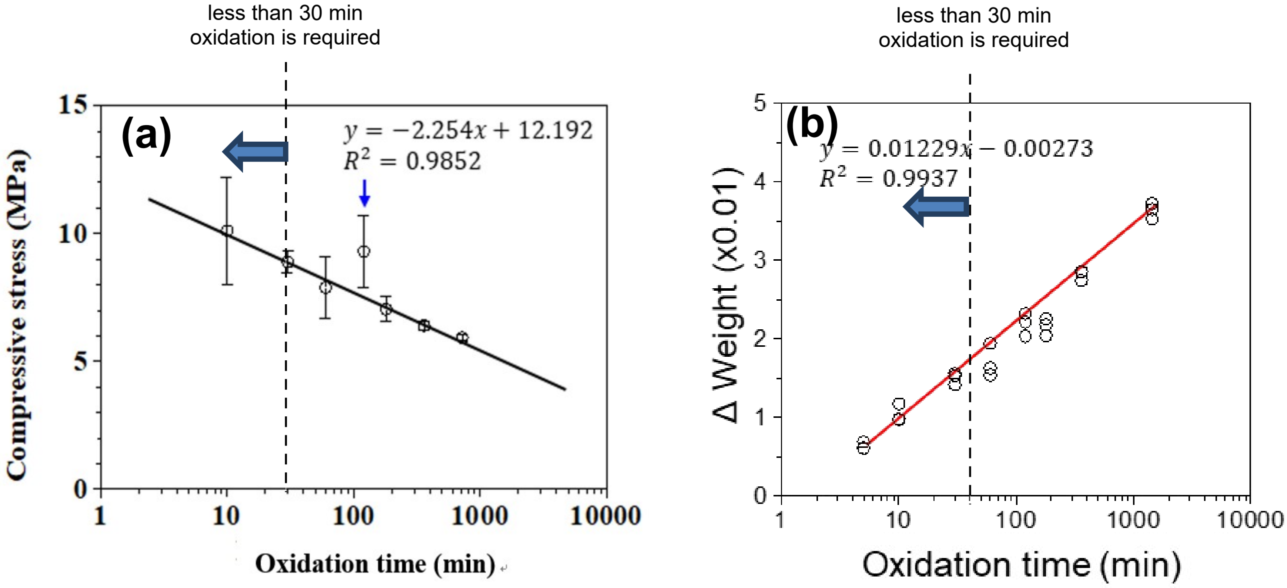

Fig. S5. (a) Compressive stress variation and (b) oxide layer weight gain of NAFA according to the oxidation time.

Table S2. Specific Gibbs free energy ($\Delta G_{specific}$) of the reaction between Li and coated materials.

Layer material	Product	$\Delta G_{specific}$ (10^9 J m^{-2})
NiO	Li_2O, Ni	$-56.1 \times t$ [A]
Ni_2O_3	Li_2O, Ni	$-59.3 \times t$ [our work]
TiO_2	Ti, Li_2O	$-8.5 \times t$[B]
ZnO	Li_3Zn, Li_2O	$-20.0 \times t$[B]
Al	Li_9Al_4	$-4.9 \times t$[B]
Au	$Li_{15}Au_4$	$-15.6 \times t$[B]
Si	$Li_{21}Si_5$	$-9.3 \times t$

Notes: t is the layer thickness.

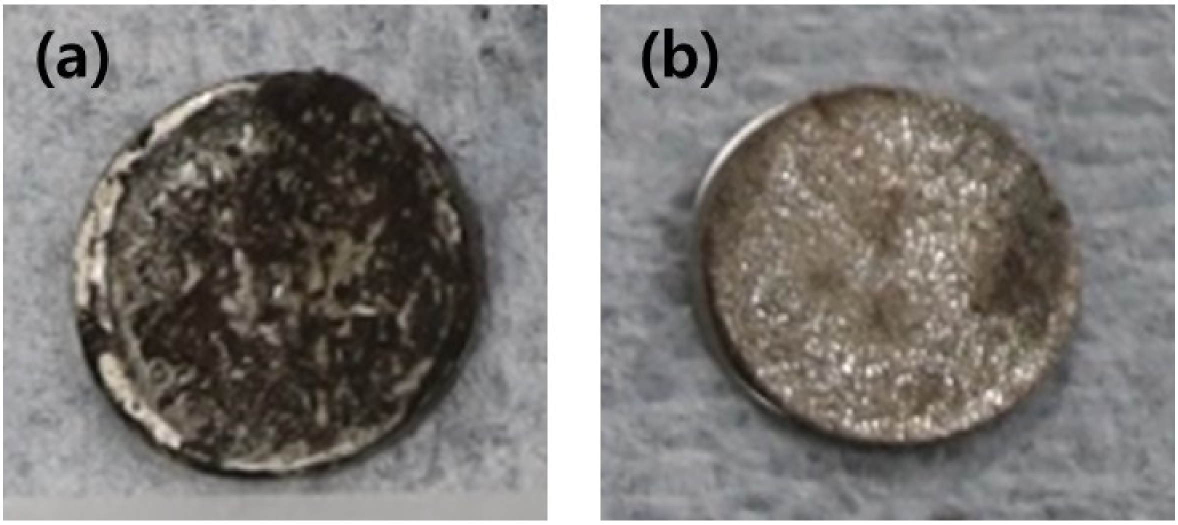

Fig. S6. Photographs of the anodes after cycle test. (a) Li-foil anode, (b) oxidised NAF anode.

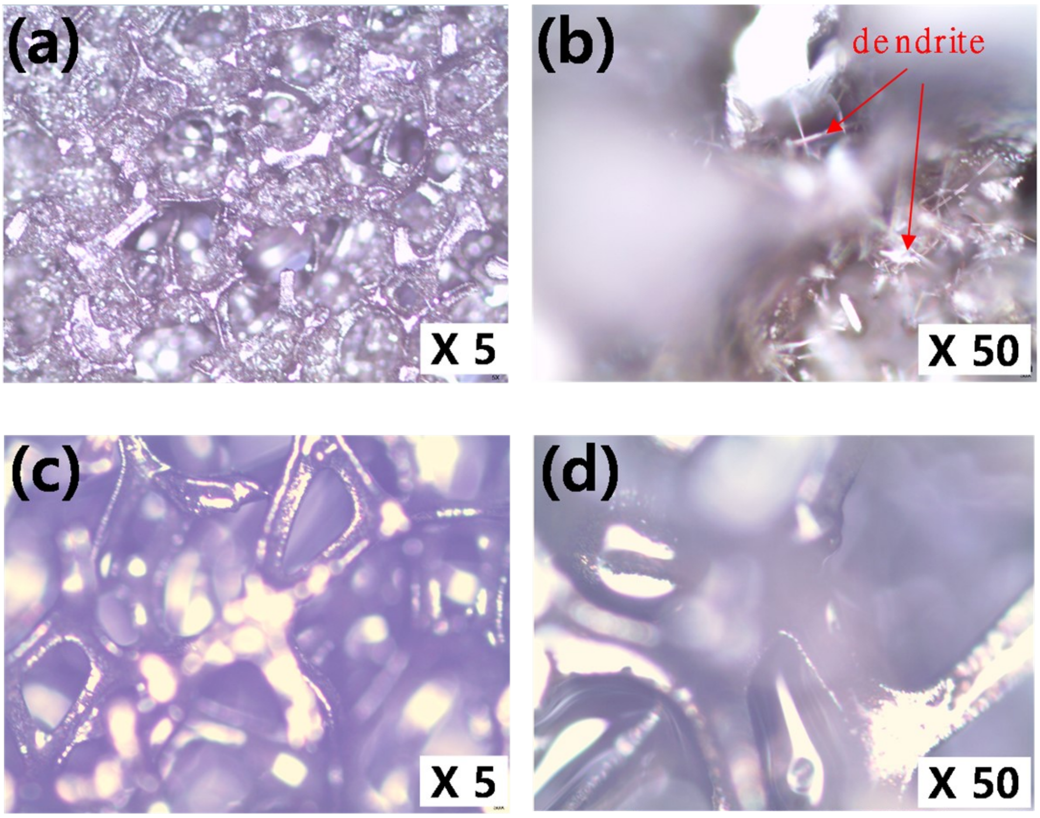

Fig. S7. Optical microscope image comparison of anodes after cycle test of the NAF control and oxidized-NAF electrodes. (a) control NAF anode at magnitude of 5 times, (b) control NAF anode at magnitude of 50 times, (c) oxidised NAF anode at magnitude of 5 times, (d) oxidised NAF anode at magnitude of 50 times.

References

[A] Choi, Y. S.; Ahn, T. Y.; Lee, E. H.; Hur, T. U.; Ha, S. H.; Lee, J. I.; Park, T. R.; Choi, C. H.; Kim, I. Y.; Cho, J. H. An ultra-lithiophilic oxidation layer in a Ni-foam-based anode for lithium metal batteries. Mater. Adv. **2021**, 2, 1972–1980. DOI: 10.1039/D1MA00065A

[B] Wang, J.; Wang, H.; Xie, J.; Yang, A.; Pei, A. Wu, C-.L.; Shi, F.; Liu, Y.; Lin, D.; Gong, Y.; Cui, Y. Fundamental study on the wetting property of liquid lithium. Energy Storage Mater. **2018**, 14, 345–350. DOI: 10.1016/j.ensm.2018.05.021
